# The Treatment of General Peritonitis

**Published:** 1907-01-12

**Authors:** 


### THE TREATMENT OF GENERAL PERITONITIS.

GENERAL peritonitis is one of those conditions the treatment of which is dependent upon its early recognition and diagnosis. Operative interference is the one means which offers any reasonable prospect of recovery, and this fact has now received general recognition. Yet Mr. Mayo Robson in a recent address on the subject still finds reason to complain that the old expectant method with administration of opium has not been fully discarded. We are confident, however, that the rank and file of the profession are not so far behind its leaders that they still adopt this method as a rational mode of treatment for acute general peritonitis. The mistake lies in a failure to recognise the early signs and symptoms of the disease and a too great anxiety to give the patient immediate relief from his suffering.

General peritonitis does not arise suddenly *per se*, labelled in plain figures, but sets in insidiously as a result of some previous abdominal affection. To relieve this primary affection opium is resorted to, and in consequence the further development of symptoms, which would lead to an immediate recognition of the spreading infection, is checked and masked, and a fatal delay results. No other morbid condition calls for such careful and exact estimation of the symptoms and the changes they may undergo. The first principle, therefore, in the treatment of these abdominal affections is the avoidance of anything like opium which may obscure the actual course of the disease. The use of purgatives is now generally condemned in these conditions. Internal peristalsis is probably largely responsible for the spread of a localised infective process to the general peritoneum. It should be, therefore, one of the aims of treatment to reduce peristalsis to a minimum. With the same idea in view food should be withheld from the stomach. Severe or continued vomiting may be controlled by lavage.

There is still some diversity in the methods of operation in cases of general peritonitis. When surgical interference was first undertaken for this condition the operator was content to relieve and if possible remedy the local lesion with as little interference, with the general abdominal cavity, as possible. So great was the respect for the peritoneal domain that the incision was not infrequently too small to allow a proper recognition of the condition of things within. Then came the bolder methods of long incisions, free explorations, attended with evisceration and irrigation of the peritoneum. It is now generally agreed that these heroic measures are not justified by the results they have yielded, and at a discussion on the subject at the last annual meeting of the British Medical Association there was a consensus of opinion in favour of a return to the more conservative methods of operation.

The researches of Sargent and Dudgeon on the bacteriology of general peritonitis have exercised a marked influence on surgical views in this respect. They showed that, with the exception of cases in which the peritoneum is suddenly overwhelmed with a virulent infection, the staphylococci albi are the first to migrate and set up a mild inflammatory reaction with increased phagocytosis and lymph formation. In this way, they suggest, protective barriers are set up against the invasion of the more virulent micro-organisms. Evisceration and irrigation, by interfering with these protective processes, render the peritoneum more liable to a subsequent general infection. But, apart from these considerations, there is the additional factor of shock, which inevitably accompanies these more extensive operations, and must exercise considerable influence on the rallying or recative powers of the patient.

Mr. Mayo Robson gives concisely the leading principles which should guide the surgeon in these operations. They are: The removal or remedy of the cause of the infection; efficient local drainage; rapidity of operation; the avoidance of unnecessary exposure and handling of viscera; and the prevention of shock.

In the after-treatment of these cases great value is now being ascribed to saline injections per rectum. Dr. Murphy, of Chicago, who is able to show a record of 35 recoveries in a series of 36 operations, lays special stress on this measure. He suggests that it reverses the direction of the lymph current, causing a secretion of fluid into the peritoneal cavity and subsequent drainage into the pelvis. It also combats shock by filling the blood-vessels in the same way as interstitial or intravenous saline injections. The semi-recumbent posture, known as Fowler's position, promotes drainage of the infective fluid from the diaphragmatic area to the less absorptive pelvic region.

By the accumulation of the results of individual experiences, a steady evolution in the surgical treatment of peritonitis has been taking place, and surgeons have learned the necessity of setting certain limitations to their interference with the natural physiological processes of the body. The striking successes, obtained by certain operators, are shown to be due to early diagnosis followed by prompt relief of the primary cause by immediate operation, and to special attention to the adjuvant measures, such as rectal saline injections. A limited surgery, combined with every effort to eliminate shock and assist the natural reactive powers of the organism to infective processes, have been attended with a greater measure of success than the more drastic methods of modern abdominal surgery.

